# Supplementary material for: Snoring and environmental exposure: results from the Swedish GA2LEN study
Source: BMJ Open. 2021 Jun 9;11(6):e044911. doi: 10.1136/bmjopen-2020-044911 (PMC8191604; doi:10.1136/bmjopen-2020-044911)

Supplemental figure 1. Directed acyclic graph of the association between snoring and environmental exposure. Green arrows indicate causal paths, while red arrows show biasing paths. http://www.dagitty.net

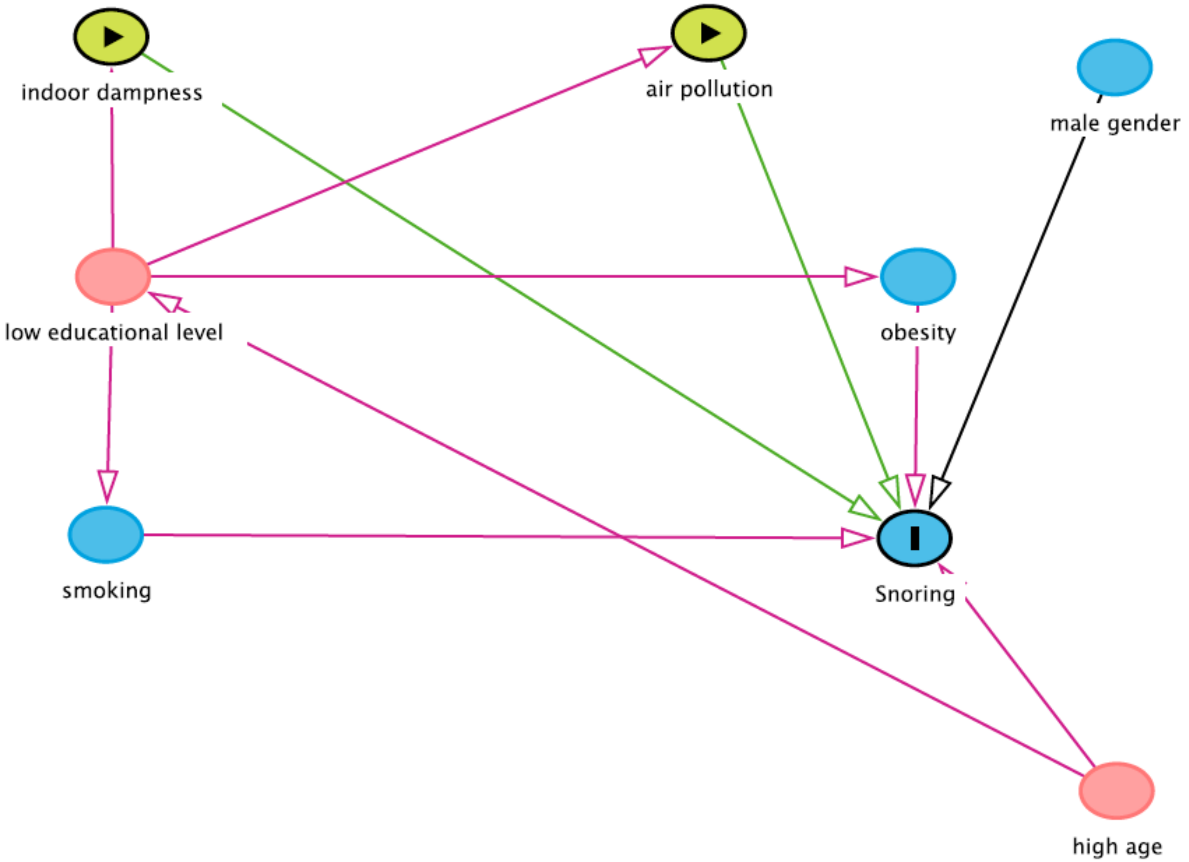

Supplement: Supplementary data [file bmjopen-2020-044911supp001.pdf]
