# Supplementary material for: Snoring and environmental exposure: results from the Swedish GA2LEN study
Source: BMJ Open. 2021 Jun 9;11(6):e044911. doi: 10.1136/bmjopen-2020-044911 (PMC8191604; doi:10.1136/bmjopen-2020-044911)
Supplement: Author's manuscript [file bmjopen-2020-044911.draft_revisions.pdf]

BMJ Open

BMJ Open is committed to open peer review. As part of this commitment we make the peer review history of every article we publish publicly available.

When an article is published we post the peer reviewers' comments and the authors' responses online. We also post the versions of the paper that were used during peer review. These are the versions that the peer review comments apply to.

The versions of the paper that follow are the versions that were submitted during the peer review process. They are not the versions of record or the final published versions. They should not be cited or distributed as the published version of this manuscript.

BMJ Open is an open access journal and the full, final, typeset and author-corrected version of record of the manuscript is available on our site with no access controls, subscription charges or pay-per-view fees (http://bmjopen.bmj.com).

If you have any questions on BMJ Open's open peer review process please email info.bmjopen@bmj.com

Snoring and environmental exposure: results from the Swedish GA2LEN study.

Journal:	*BMJ Open*
Manuscript ID	bmjopen-2020-044911
Article Type:	Original research
Date Submitted by the Author:	24-Sep-2020

Complete List of Authors:	Silverforsen, Daniel; Uppsala University, Dep of Medical Sciences: Respiratory Medicine<br>Theorell-Haglöw, J; Uppsala University, Department of Medical Sciences<br>Ljunggren, Mirjam; Uppsala Univ<br>Middelveld, Roelinde<br>Wang, Juan; Uppsala University<br>Franklin, Karl; Umeå University Hospital, Department of Surgery<br>Norbäck, Dan; Uppsala universitet Institutionen for medicinska vetenskaper, Occupational and Environmental Medicine<br>Lundbäck, Bo; Goteborgs Universitet, Krefting Research Centre<br>Forsberg, Bertil; Umeå University, Public Health and Clinical Medicine<br>Lindberg, Eva ; Uppsala University, Dep om Medical Sciences: Respiratory Medicine<br>Janson, Christer; Uppsala University, Dep of Medical Sciences: Respiratory Medicine

Keywords:	SLEEP MEDICINE, Epidemiology < THORACIC MEDICINE, PUBLIC HEALTH

SCHOLARONE™ Manuscripts

I, the Submitting Author has the right to grant and does grant on behalf of all authors of the Work (as defined in the below author licence), an exclusive licence and/or a non-exclusive licence for contributions from authors who are: i) UK Crown employees; ii) where BMJ has agreed a CC-BY licence shall apply, and/or iii) in accordance with the terms applicable for US Federal Government officers or employees acting as part of their official duties; on a worldwide, perpetual, irrevocable, royalty-free basis to BMJ Publishing Group Ltd ("BMJ") its licensees and where the relevant Journal is co-owned by BMJ to the co-owners of the Journal, to publish the Work in this journal and any other BMJ products and to exploit all rights, as set out in our licence.

The Submitting Author accepts and understands that any supply made under these terms is made by BMJ to the Submitting Author unless you are acting as an employee on behalf of your employer or a postgraduate student of an affiliated institution which is paying any applicable article publishing charge ("APC") for Open Access articles. Where the Submitting Author wishes to make the Work available on an Open Access basis (and intends to pay the relevant APC), the terms of reuse of such Open Access shall be governed by a Creative Commons licence – details of these licences and which Creative Commons licence will apply to this Work are set out in our licence referred to above.

Other than as permitted in any relevant BMJ Author's Self Archiving Policies, I confirm this Work has not been accepted for publication elsewhere, is not being considered for publication elsewhere and does not duplicate material already published. I confirm all authors consent to publication of this Work and authorise the granting of this licence.

For peer review only

Snoring and environmental exposure: results from the Swedish GA2LEN study.

Daniel Silverforsen[1], Jenny Theorell Haglöw[1], Mirjam Ljunggren[1], Roelinde Middelweld[2], Juan Wang[3], Karl Franklin[4], Dan Norbäck[3], Bo Lundbäck[5], Bertil Forsberg[6], Eva Lindberg[1], Christer Janson[1]

1. Department of Medical Sciences: Respiratory, Allergy and Sleep Research, Uppsala University, Uppsala, Sweden.
2. The Centre for Allergy Research, Karolinska Institutet and The Institute of Environmental Medicine, Karolinska Institutet, Stockholm, Sweden
3. Department of Medical Sciences: Occupational and Environmental Medicine, Uppsala University, Uppsala, Sweden.
4. Department of Surgery, Umeå University, Umeå, Sweden
5. Department of Internal Medicine, Krefting Research Center, Sahlgrenska Academy, University of Gothenburg, Gothenburg, Sweden
6. Occupational and Environmental Medicine, Department of Public Health and Clinical Medicine, Umeå University, Umeå, Sweden

For peer review only

For peer review only - http://bmjopen.bmj.com/site/about/guidelines.xhtml

Abstract:

Objective: Habitual snoring is associated with fatigue, headaches, and low work performance. This cross sectional study aimed to investigate if snoring is affected by environmental factors such as home dampness and exposure to air pollution.

Methods: We used data collected from the Swedish part of the epidemiological GA2LEN study carried out in 2008. The participants completed a postal questionnaire on snoring and, indoor and outdoor environmental exposure as well as potential confounders including smoking, weight, height and educational level.

Results: Of the 25,848 participants, 4,211 (16.3%) were habitual snorers. Habitual snorers reported water damage (8.3% vs. 7.0% p<0.0001), floor dampness (4.6% vs. 3.8%% p<0.0001) and visible mould (5.2% vs. 3.8% p<0.0001) in their homes more often than non-snorers. Habitual snorers stated being annoyed by air pollution more often than non-snorers with habitual snorers reporting being irritated with the air in their residential area to a higher extent (*sometimes* 16.2% vs.13.9%, and *daily* 4.6% vs.3.1%) as well as annoyance from traffic fumes (*somewhat* 19% vs.18.5% and *very* 5% vs 3.6%) (p<0.0001). These results remained significant after adjustment for age, sex, BMI, smoking history and educational level.

Conclusion: Snoring is more prevalent in subjects reporting home dampness and air pollution. This further strengthens the importance of environmental factors on health.

For peer review only

Strength and limitations

The sample size is large giving it considerate power and the possibility to simultaneously control for several confounding risk factors.

The association between snoring and both indoor and outdoor exposure has only been studied to a limted extent previously

The study is cross sectional and causalty can therefore not been assessed.

The assessments of exposure are self-reported and we know that there is only a moderate association between self-reported and measured air pollution.

For peer review only

For peer review only - http://bmjopen.bmj.com/site/about/guidelines.xhtml

Introduction

Snoring is a sign of compromised upper airway and a common symptom in obstructive sleep apnoea (OSA).[1] Snoring is a health problem also in the absence of sleep apnoea, and studies in both adults and children show that it is the frequency of snoring rather than the apnoea hypopnea index (AHI) that predicts symptoms and poorer behavioural and cognitive outcomes.[2, 3]

Habitual snoring is reported to occur in between 16-33% in men and 6-19% in women. [4-8] Male sex, age, obesity, smoking and nasal congestion are all well-established risk factors for habitual snoring. [9-14] An association between home dampness and insomnia symptoms was reported in a population-based cross-sectional study,[15] but only a few studies have, to our knowledge investigated the possible association between habitual snoring and building dampness [16].

Air pollutants such as particulate matter (PM), ozone (O_3) and nitrogen dioxide (NO_2) all have adverse effects on the airways. [17] An association between exposure to air pollutants and snoring have been reported in children [18, 19] and habitual snoring was associated with having a bedroom with traffic noise but not to high exposure to traffic pollution in a Nordic population study .[20] In another study, exposure to ozone was associated with an increase in AHI.[21]

The aim of this study was to investigate if habitual snoring is affected by environmental factors. We hypothesised that both exposures to perceived building dampness and air pollution are associated with habitual snoring.

For peer review only - http://bmjopen.bmj.com/site/about/guidelines.xhtml

Method

The Global Asthma and Allergy and European network of excellence (GA²LEN) was established 2004 as a consortium in the field of asthma and allergy.[22] The GA²LEN epidemiological study was carried out in 2008 across Europe, and the aim was to assess the prevalence of allergic disease and its associated risk factors. [23] The present investigation was based on the Swedish part of the postal survey carried out in four centres; Stockholm, Gothenburg, Uppsala and Umeå. A random sample of 15,000 subjects from Gothenburg and 10000 from each of the other cities was selected, and the participants were 16-75 years old.[24] The questionnaire included questions on asthma, chronic bronchitis, eczema, insomnia, OSA, physical activity and environmental and workplace exposure. Up to three reminders were mailed, and 26,577 subjects completed the questionnaire. In the present analysis, we included the 25,848 participants who had answered both the question regarding snoring and environmental exposure.

Definitions:

Snoring was assessed by the question "in the last few months, how many times have you snored loudly and disturbingly". Habitual snorers were defined as having answered either, 3-5 times per week or every or almost every night.

Smoking was assessed by the questions "Have you ever smoked one or more cigarettes per day for more than one year?" and "Have you smoked at all during the last month?".

Current smoking was defined as positive responses to the questions "Have you ever smoked for at least one year?" and "Have you smoked at all during the last month?".

Previous smoking was defined as a positive response to the question "Have you ever smoked for at least one year?" and a negative response to the question "Have you smoked at all during the last month?".

Never smoking was defined as a negative response to the question "Have you ever smoked for at least one year?".

Perceived annoyance due to air pollution was assessed by two question "Do you find the air irritating in your residential area: 1, Daily/almost daily (Daily) 2, Sometimes/periodically (Sometimes) 3, Seldom/never (Never) and "How annoyed are you by fumes from traffic in your residential area.": 1, None/a little (None) 2, Somewhat 3 Very much.

Dampness was defined as a positive response to at least one of the following questions: "Have any of the following been noted in your home during the last 12 months:

(a) Water-/moisture damage on indoor walls, floors or ceiling? (Water damage)

For peer review only - http://bmjopen.bmj.com/site/about/guidelines.xhtml

(b) Dented plastic mats, yellowed plastic mats or blackened parquet? (Floor dampness)
(c) Visible moulds on walls, floors or ceiling?" (Visible moulds)

Educational level was assessed, and the subjects were divided into three groups depending on the highest academic level: primary school, high school, university.

Body mass index (BMI) was calculated from the subjects answer to their height and weight.

Statistical Analysis:
The statistics where analysed using STATA version 15 (STATA Corp, College Station, Texas, USA). Chi-squared test and unpaired t-test were used in unadjusted analyses when comparing habitual snorers and non-snorers. Multiple logistic regression analysis was performed when studying the relationship between habitual snoring and environmental exposure after having adjusted for the confounding factors age, sex, BMI, smoking history and educational level. Test for interaction was done for building dampness and air pollution indicators in the relationship with snoring. We also used test for interaction to determine if there was a sex difference in the association between snoring and environmental variables. A p value of <0.05 was considered to be statistically significant.

For peer review only

Results

Of the 25,848 participants 4,211 (16.3%) were habitual snorers. Habitual snoring was more common in men and in older participants (Table 1). Habitual snorers had a higher mean BMI, were less likely to have finished higher education and were more likely to be ex- and current smokers compared with the non-snorers (Table 1).

Habitual snorers reported water damage, floor dampness and visible mould more often than non-snorers (Table 2). The habitual snorers also stated being annoyed by air pollution more often than non-snorers (Table 2).

Residential water damage (OR (95% CI)) 1.29 (1.13-1.48)), floor dampness (OR 1.21 (1.01-1.45)) and visible moulds (OR 1.51 (1.27-1.80)) were significantly associated with habitual snoring also after adjustment for confounders (age, sex, BMI, smoking history and educational level) (Figure1). There was also an association between being sometimes and daily annoyed by air pollution in the residential area (OR 1.30 (1.17-1.43) and 1.64 (1.36-1.98)), respectively) and by somewhat and daily annoyed by traffic and habitual snoring (OR 1.15 (1.04-1.26) and 1.51 (1.27-1.80)), respectively that remained statistically significant after adjustment for confounders (Figure 1).

There was no significant interaction for building dampness and air pollution in the relationship with snoring or between exposure variables and sex regarding the outcomes (all p-values > 0.05).

For peer review only - http://bmjopen.bmj.com/site/about/guidelines.xhtml

Discussion

The main result of this study was that both home dampness and self-reported exposure to air pollution was significantly associated with habitual snoring. The results remained significant after having adjusted for age, sex, BMI, smoking history and educational level.

Of the indoor dampness indicators, visible mould had the highest odds ratio for habitual snoring followed by water damage and floor dampness. As far as we know, a relationship between home dampness and snoring has only been been in a few studies.[16] Previous studies on home dampness have looked at other respiratory disorders and shown associations to conditions such as asthma and chronic rhinosinusitis. [25-27] Zhang et al 2019 showed an association for non-respiratory symptoms such as eye and skin symptoms, headache, and fatigue. [28] An association between indoor dampness and insomnia symptoms has also been reported. [15] Headache and fatigue are also symptoms that are associated with snoring. [10, 29] It is possible that the association between home dampness, fatigue and headache partly is related to a higher incidence of habitual snoring in individuals living in damp homes.

The association between exposure to air pollution and snoring in adults found in our study is somewhat in accordance with previous research by Weinreich *et al* [21]. In that study an interquartile range increase in ozone was associated with a 10.1% increase in AHI. In a study by Kheirandish-Gozal *et al* [18] children residing in the neighbourhoods with the poorest air quality had a significantly higher frequency of habitual snoring compared to those who lived the neighbourhoods with the lowest degrees of air pollution.[18] The association to habitual snoring are, however, not in line with the findings of Gislason et.al [20] who studied self-reported traffic exposure and its association with habitual snoring. In that study traffic noise but not high air pollution exposure was associated with habitual snoring. An association between air pollution and habitual snoring seems plausible since both passive and active smoking has been shown to be associated with habitual snoring. [13] Air pollution and cigarette smoke share some chemical components and mechanic pathways. [30]

The prevalence of habitual snoring was 16% in our study sample. This is lower compared to a similar study on a Scandinavian population, which showed a prevalence of 25%. [20] The study used the same question and criteria when classifying habitual snoring, however the questionnaire was sent to subjects in both Sweden, Estonia, Iceland, Denmark and Norway and the different geographical locations of the participants and the older population might explain the difference in prevalence. Smoking, BMI, physical activity, socioeconomic background and other risk factors to

For peer review only - http://bmjopen.bmj.com/site/about/guidelines.xhtml

snoring varies widely depending on geographical location. Habitual snoring was also associated with older age, male sex, higher BMI and smoking in our study. This is in line with previous research and are probably the more well studied risk factors for snoring. [9]

The biological explanation for the association between snoring and environmental exposure in our study is not completely known. Home dampness and exposure to airway pollution has been connected with inflammatory markers in nasal lavage.[31, 32] It is therefore possible that inflammation in the upper airways caused by home dampness and air pollution explains the association to snoring.

The study uses a large sample size, consisting of 25,848 subjects from different parts of Sweden, thereby giving it considerate power and the possibility to simultaneously control for several confounding risk factors. However, there are weaknesses to be considered. The assessments of exposure are self-reported and we know that there is only a moderate association between self-reported and measured air pollution.[33] It is difficult to say how accurate the subjects' assessment of their own snoring habits are and it has been shown that there are some discrepancies between self-reported and spouse-reported snoring habits. [34] We have also no data on if the subjects live by themselves or not, a factor which may affect individuals knowledge of their snoring.

Our results on airway pollution and home dampness association with habitual snoring warrant further studies in the area. These results could further implicate the importance of good air quality in home environment. Since snoring is a common occurrence and has been associated with negative health effects limiting risk factors might have a health economic impact, adding to the importance of good residential building codes. Future studies in the area, such as a follow-up study on snorers moving from areas with high air pollution to lower air pollution or from homes with signs of dampness to homes without would be interesting.

In conclusion, habitual snoring is a common occurrence associated with several negative health effects. Home dampness as well as exposure to air pollution was significantly associated with habitual snoring after having adjusted for cofounders. This further strengthens the importance of environmental factors on health.

For peer review only - http://bmjopen.bmj.com/site/about/guidelines.xhtml

Acknowledgements

The study was carried out as part of the GA2LEN survey and follow-up and was supported by the EU FP6 project GA2LEN (EU contract nr. FOOD-CT-2004-506378).

For peer review only - http://bmjopen.bmj.com/site/about/guidelines.xhtml

References

1. Zhang, W. and L.Y. Si, *Obstructive sleep apnea syndrome (OSAS) and hypertension: pathogenic mechanisms and possible therapeutic approaches.* Ups J Med Sci, 2012. **117**(4): p. 370-82.
2. Smith, D.L., et al., *Frequency of snoring, rather than apnea-hypopnea index, predicts both cognitive and behavioral problems in young children.* Sleep Med, 2017. **34**: p. 170-178.
3. Svensson, M., et al., *Daytime sleepiness relates to snoring independent of the apnea-hypopnea index in women from the general population.* Chest, 2008. **134**(5): p. 919-924.
4. Enright, P.L., et al., *Prevalence and correlates of snoring and observed apneas in 5,201 older adults.* Sleep, 1996. **19**(7): p. 531-8.
5. Gislason, T., et al., *Prevalence of sleep apnea syndrome among Swedish men--an epidemiological study.* J Clin Epidemiol, 1988. **41**(6): p. 571-6.
6. Kayukawa, Y., et al., *Habitual snoring in an outpatient population in Japan.* Psychiatry Clin Neurosci, 2000. **54**(4): p. 385-91.
7. Lindberg, E., et al., *A 10-year follow-up of snoring in men.* Chest, 1998. **114**(4): p. 1048-55.
8. Svensson, M., *Risk Factors Associated With Snoring in Women With Special Emphasis on Body Mass Index: A Population-Based Study.* Chest, 2006. **129**(4): p. 933-941.
9. Bloom, J.W., W.T. Kaltenborn, and S.F. Quan, *Risk factors in a general population for snoring. Importance of cigarette smoking and obesity.* Chest, 1988. **93**(4): p. 678-83.
10. Janson, C., et al., *Daytime sleepiness, snoring and gastro-oesophageal reflux amongst young adults in three European countries.* J Intern Med, 1995. **237**(3): p. 277-85.
11. Palm, A., C. Janson, and E. Lindberg, *The impact of obesity and weight gain on development of sleep problems in a population-based sample.* Sleep Med, 2015. **16**(5): p. 593-7.
12. Wali, S.O. and B.A. Abaalkhail, *Prevalence and predictors of habitual snoring in a sample of Saudi middle-aged adults.* Saudi Med J, 2015. **36**(8): p. 920-7.
13. Franklin, K.A., et al., *The influence of active and passive smoking on habitual snoring.* Am J Respir Crit Care Med, 2004. **170**(7): p. 799-803.
14. Young, T., L. Finn, and M. Palta, *Chronic nasal congestion at night is a risk factor for snoring in a population-based cohort study.* Arch Intern Med, 2001. **161**(12): p. 1514-9.
15. Janson, C., et al., *Insomnia is more common among subjects living in damp buildings.* Occup Environ Med, 2005. **62**(2): p. 113-8.
16. Wang, J., et al., *Dampness and mold at home and at work and onset of insomnia symptoms, snoring and excessive daytime sleepiness.* Environ Int, 2020. **139**: p. 105691.
17. Guan, W.J., et al., *Impact of air pollution on the burden of chronic respiratory diseases in China: time for urgent action.* Lancet, 2016. **388**(10054): p. 1939-1951.
18. Kheirandish-Gozal, L., et al., *Neighbourhood air quality and snoring in school-aged children.* Eur Respir J, 2014. **43**(3): p. 824-32.
19. Sanchez, T., et al., *Association between air pollution and sleep disordered breathing in children.* Pediatr Pulmonol, 2019. **54**(5): p. 544-550.
20. Gislason, T., et al., *Self-reported exposure to traffic pollution in relation to daytime sleepiness and habitual snoring: a questionnaire study in seven North-European cities.* Sleep Med, 2016. **24**: p. 93-99.
21. Weinreich, G., et al., *Association of short-term ozone and temperature with sleep disordered breathing.* Eur Respir J, 2015. **46**(5): p. 1361-9.
22. Van Cauwenberge, P., et al., *Spreading excellence in allergy and asthma: the GA2LEN (Global Allergy and Asthma European Network) project.* Allergy, 2005. **60**(7): p. 858-864.
23. Jarvis, D., et al., *Asthma in adults and its association with chronic rhinosinusitis: the GA2LEN survey in Europe.* Allergy, 2012. **67**(1): p. 91-8.
24. Sundbom, F., et al., *Asthma symptoms and nasal congestion as independent risk factors for insomnia in a general population: results from the GA(2)LEN survey.* Allergy, 2013. **68**(2): p. 213-9.
25. Ahlroth Pind, C., et al., *Patient-reported signs of dampness at home may be a risk factor for chronic rhinosinusitis: A cross-sectional study.* Clin Exp Allergy, 2017. **47**(11): p. 1383-1389.
26. Gunnbjornsdottir, M.I., et al., *Indoor environment in three North European cities in relationship to atopy and respiratory symptoms.* Clin Respir J, 2009. **3**(2): p. 85-94.

For peer review only - http://bmjopen.bmj.com/site/about/guidelines.xhtml

27. Wang, J., et al., *Dampness, mould, onset and remission of adult respiratory symptoms, asthma and rhinitis.* Eur Respir J, 2019. **53**(5).
28. Zhang, X., et al., *Dampness and mold in homes across China: Associations with rhinitis, ocular, throat and dermal symptoms, headache and fatigue among adults.* Indoor Air, 2019. **29**(1): p. 30-42.
29. Stark, C.D. and R.J. Stark, *Sleep and chronic daily headache.* Curr Pain Headache Rep, 2015. **19**(1): p. 468.
30. Ghio, A.J., J.M. Soukup, and M.C. Madden, *The toxicology of air pollution predicts its epidemiology.* Inhalation Toxicology, 2018. **30**(9-10): p. 327-334.
31. Chen, B.Y., et al., *The association of ambient air pollution with airway inflammation in schoolchildren.* Am J Epidemiol, 2012. **175**(8): p. 764-74.
32. Wieslander, G., et al., *Nasal and ocular symptoms, tear film stability and biomarkers in nasal lavage, in relation to building-dampness and building design in hospitals.* Int Arch Occup Environ Health, 1999. **72**(7): p. 451-61.
33. Carlsen, H.K., et al., *Indicators of residential traffic exposure: Modelled NOX, traffic proximity, and self-reported exposure in RHINE III.* Atmospheric Environment, 2017. **167**: p. 416-425.
34. Wiggins, C.L., et al., *Comparison of self- and spouse reports of snoring and other symptoms associated with sleep apnea syndrome.* Sleep, 1990. **13**(3): p. 245-52.

For peer review only

For peer review only - http://bmjopen.bmj.com/site/about/guidelines.xhtml

Contributors CJ is the correspondence author and DS and CJ drafted the paper and performed the statically analysis, CJ, RM, KF, BF and BL participated in designing the study and reviewed the manuscript; JT, MJ, RM, JW, KG, DN. BL, BF and EL also reviewed the paper on several stages

Funding This work has been supported by supported by the EU FP6 project GA2LEN (EU contract nr. FOOD-CT-2004-506378), the Centre for Allergy Research at the Karolinska Institutet, the Swedish Heart Lung Foundation, the Swedish Heart and Lung Association and the Swedish Asthma and Allergy Association.

Competing interests None declared.

Patient and public involvement No patient involved

Patient consent for publication Not required.

Ethics approval The study was approved by the Regional Ethical Review Board in Uppsala, Sweden approval (Dnr 2008/014).

Data availability statement Data are available upon reasonable request. Requests for data should be sent to the corresponding author.

For peer review only

Table 1. Characteristics of non-snorers and snorers (% and mean±SD)

	Non-snorers (n=21,637)	Habitual snores (n=4,211)	p-value
Men	42.3	62.6	<0.0001
Age	42.1±16.0	50.0±13.9	<0.0001
Smoking history			<0.0001
never	63.7	45.7	
ex	23.6	34.2	
current	12.7	20.1	
BMI	24.3±3.9	27.1±4.6	<0.0001
Educational level			<0.0001
Primary school,	12.4	18.5	
Secondary school	28.5	35.8	
Tertiary School	59.1	45.8	

Table 2. Environmental exposure in non-snorers and snorers (%)

	Non-snorers (n=21,637)	Habitual snores (4,211)	p-value
Indoor dampness			
Water damage	7.0	8.3	0.002
Floor dampness	3.8	4.6	0.02
Visible moulds	3.8	5.2	<0.0001
Annoyed by air pollution in residential area			<0.0001
Never	83.0	79.2	
Sometimes	13.9	16.2	
Daily	3.1	4.6	
Annoyed by air pollution from traffic			<0.0001
None	77.9	76.0	
Somewhat	18.5	19.0	
Very much	3.6	5.0	

For peer review only

Figure 1. Association between environmental exposure and habitual snoring (adjusted odds ratio (adj. OR) and 95% confidence interval (CI))

* adjusted for age, sex, BMI, smoking history, educational level and residential air pollution
** adjusted for age, sex, BMI, smoking history, educational level and residential water damage

For peer review only

For peer review only - http://bmjopen.bmj.com/site/about/guidelines.xhtml

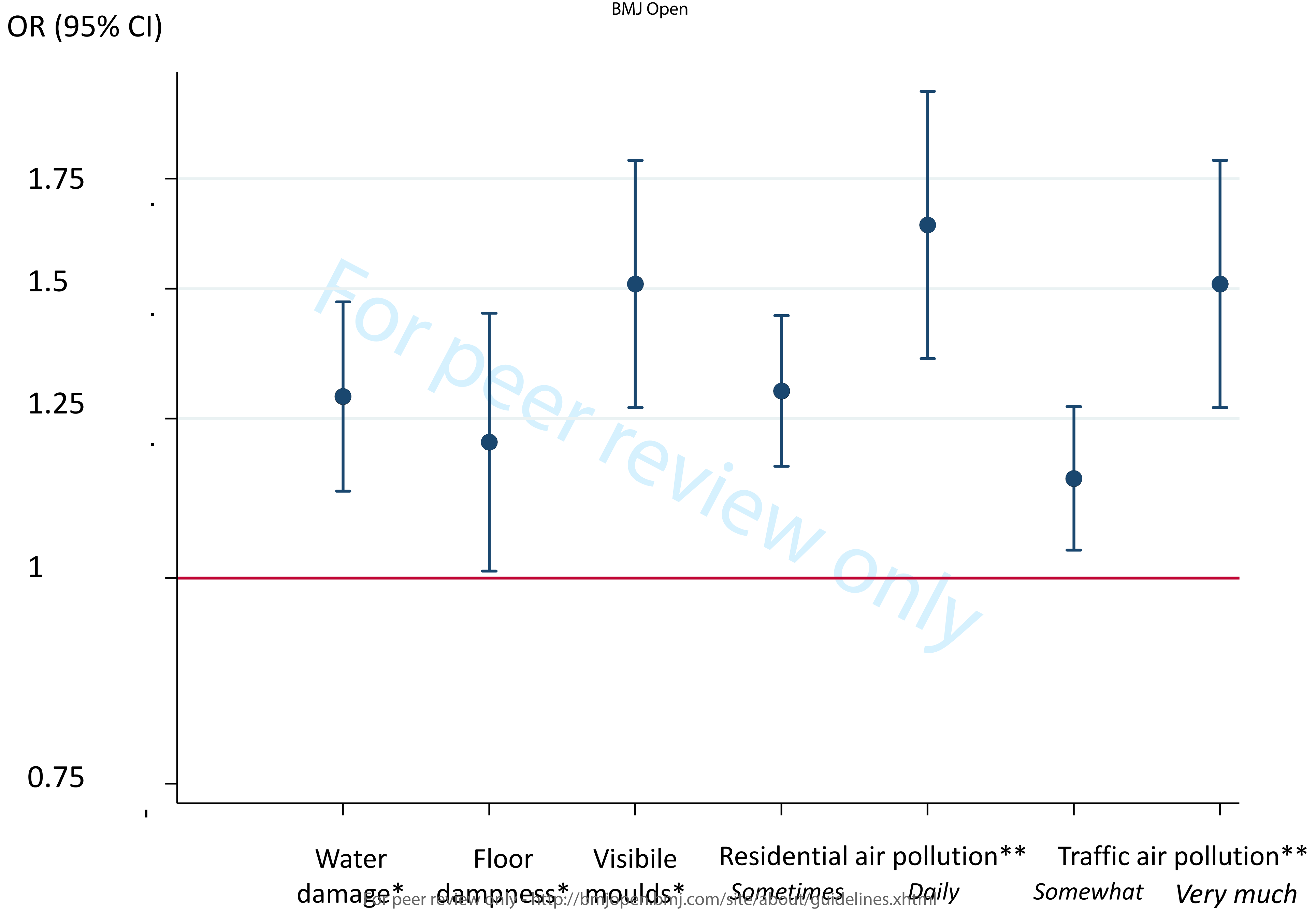

For peer review only

STROBE Statement—Checklist of items that should be included in reports of ***cross-sectional studies***

	Item Snoring and environmental exposure: results from the Swedish GA2LEN study No	Recommendation
**Title and abstract**	1	(*a*) Indicate the study's design with a commonly used term in the title or the abstract<br>Page 2, paragraph 1
		(*b*) Provide in the abstract an informative and balanced summary of what was done and what was found
**Introduction**		
Background/rationale	2	Explain the scientific background and rationale for the investigation being reported<br>Page 4, paragraph 1-3
Objectives	3	State specific objectives, including any prespecified hypotheses<br>Page 4, paragraph 4
**Methods**		
Study design	4	Present key elements of study design early in the paper<br>Page 5, paragraph 1
Setting	5	Describe the setting, locations, and relevant dates, including periods of recruitment, exposure, follow-up, and data collection<br>Page 5, paragraph 1
Participants	6	(*a*) Give the eligibility criteria, and the sources and methods of selection of participant<br>Page 5, paragraph 1s
Variables	7	Clearly define all outcomes, exposures, predictors, potential confounders, and effect modifiers. Give diagnostic criteria, if applicable<br>Page 5, paragraph 2- page 6, paragraph 3
Data sources/ measurement	8*	For each variable of interest, give sources of data and details of methods of assessment (measurement). Describe comparability of assessment methods if there is more than one group<br>Page 5, paragraph 2- page 6, paragraph 3
Bias	9	Describe any efforts to address potential sources of bias<br>Not relevant
Study size	10	Explain how the study size was arrived at<br>Page 5, paragraph 1
Quantitative variables	11	Explain how quantitative variables were handled in the analyses. If applicable, describe which groupings were chosen and why<br>Page 6, paragraph 4
Statistical methods	12	(*a*) Describe all statistical methods, including those used to control for confounding<br>Page 6, paragraph 4
		(*b*) Describe any methods used to examine subgroups and

For peer review only - http://bmjopen.bmj.com/site/about/guidelines.xhtml

		interactions<br>Page 6, paragraph 4
		(*c*) Explain how missing data were addressed
		(*d*) If applicable, describe analytical methods taking account of sampling strategy
		(*e*) Describe any sensitivity analyses
**Results**		
Participants	13*	(a) Report numbers of individuals at each stage of study—eg numbers potentially eligible, examined for eligibility, confirmed eligible, included in the study, completing follow-up, and analysed<br>Page 5, paragraph 1 and Page 7, paragraph 1
		(b) Give reasons for non-participation at each stage<br>Page 5, paragraph 1
		(c) Consider use of a flow diagram<br>Will add little of value in this paper
Descriptive data	14*	(a) Give characteristics of study participants (eg demographic, clinical, social) and information on exposures and potential confounders<br>Table 1
		(b) Indicate number of participants with missing data for each variable of interest
Outcome data	15*	Report numbers of outcome events or summary measures<br>Page 5, paragraph 1
Main results	16	(*a*) Give unadjusted estimates and, if applicable, confounder-adjusted estimates and their precision (eg, 95% confidence interval). Make clear which confounders were adjusted for and why they were included<br>Table 2, Figure 1, Page 7, paragraph 3
		(*b*) Report category boundaries when continuous variables were categorized<br>Not relevant
		(*c*) If relevant, consider translating estimates of relative risk into absolute risk for a meaningful time period<br>Not relevant
Other analyses	17	Report other analyses done—eg analyses of subgroups and interactions, and sensitivity analyses<br>Page 7, paragraph 4
**Discussion**		
Key results	18	Summarise key results with reference to study objectives<br>Page 8, paragraph 1
Limitations	19	Discuss limitations of the study, taking into account sources of potential bias or imprecision. Discuss both direction and magnitude of any potential bias<br>Page 9, paragraph 3
Interpretation	20	Give a cautious overall interpretation of results considering objectives, limitations, multiplicity of analyses, results

For peer review only

		from similar studies, and other relevant evidence<br>Page 9, paragraph 4 and 5
Generalisability	21	Discuss the generalisability (external validity) of the study results<br>Page 9, paragraph 4 and 5
**Other information**		
Funding	22	Give the source of funding and the role of the funders for the present study and, if applicable, for the original study on which the present article is based<br>Page 13

*Give information separately for exposed and unexposed groups.

Note: An Explanation and Elaboration article discusses each checklist item and gives methodological background and published examples of transparent reporting. The STROBE checklist is best used in conjunction with this article (freely available on the Web sites of PLoS Medicine at http://www.plosmedicine.org/, Annals of Internal Medicine at http://www.annals.org/, and Epidemiology at http://www.epidem.com/). Information on the STROBE Initiative is available at www.strobe-statement.org.

For peer review only

For peer review only - http://bmjopen.bmj.com/site/about/guidelines.xhtml

BMJ Open

Snoring and environmental exposure: results from the Swedish GA2LEN study.

Journal:	*BMJ Open*
Manuscript ID	bmjopen-2020-044911.R1
Article Type:	Original research
Date Submitted by the Author:	01-Mar-2021
Complete List of Authors:	Silverforsen, Daniel; Uppsala Universitet, Dep of Medical Sciences: Respiratory Medicine<br>Theorell-Haglöw, J; Uppsala Universitet, Department of Medical Sciences<br>Ljunggren, Mirjam; Uppsala Universitet<br>Middelveld, Roelinde<br>Wang, Juan; Uppsala University<br>Franklin, Karl; Umea Universitet, Department of Surgery<br>Norbäck, Dan; Uppsala universitet Institutionen for medicinska vetenskaper, Occupational and Environmental Medicine<br>Lundbäck, Bo; Goteborgs Universitet, Krefting Research Centre<br>Forsberg, Bertil; Umea Universitet, Public Health and Clinical Medicine<br>Lindberg, Eva ; Uppsala Universitet, Dep om Medical Sciences: Respiratory Medicine<br>Janson, Christer; Uppsala Universitet, Dep of Medical Sciences: Respiratory Medicine
**Primary Subject Heading**:	Respiratory medicine
Secondary Subject Heading:	Epidemiology
Keywords:	SLEEP MEDICINE, Epidemiology < THORACIC MEDICINE, PUBLIC HEALTH

SCHOLARONE™
Manuscripts

I, the Submitting Author has the right to grant and does grant on behalf of all authors of the Work (as defined in the below author licence), an exclusive licence and/or a non-exclusive licence for contributions from authors who are: i) UK Crown employees; ii) where BMJ has agreed a CC-BY licence shall apply, and/or iii) in accordance with the terms applicable for US Federal Government officers or employees acting as part of their official duties; on a worldwide, perpetual, irrevocable, royalty-free basis to BMJ Publishing Group Ltd ("BMJ") its licensees and where the relevant Journal is co-owned by BMJ to the co-owners of the Journal, to publish the Work in this journal and any other BMJ products and to exploit all rights, as set out in our licence.

The Submitting Author accepts and understands that any supply made under these terms is made by BMJ to the Submitting Author unless you are acting as an employee on behalf of your employer or a postgraduate student of an affiliated institution which is paying any applicable article publishing charge ("APC") for Open Access articles. Where the Submitting Author wishes to make the Work available on an Open Access basis (and intends to pay the relevant APC), the terms of reuse of such Open Access shall be governed by a Creative Commons licence – details of these licences and which Creative Commons licence will apply to this Work are set out in our licence referred to above.

Other than as permitted in any relevant BMJ Author's Self Archiving Policies, I confirm this Work has not been accepted for publication elsewhere, is not being considered for publication elsewhere and does not duplicate material already published. I confirm all authors consent to publication of this Work and authorise the granting of this licence.

For peer review only

Snoring and environmental exposure: results from the Swedish GA2LEN study.

Daniel Silverforsen[1], Jenny Theorell Haglöw[1], Mirjam Ljunggren[1], Roelinde Middelveld[2], Juan Wang[3], Karl Franklin[4], Dan Norbäck[3], Bo Lundbäck[5], Bertil Forsberg[6], Eva Lindberg[1], Christer Janson[1]

1. Department of Medical Sciences: Respiratory, Allergy and Sleep Research, Uppsala University, Uppsala, Sweden.
2. The Centre for Allergy Research, Karolinska Institutet and The Institute of Environmental Medicine, Karolinska Institutet, Stockholm, Sweden
3. Department of Medical Sciences: Occupational and Environmental Medicine, Uppsala University, Uppsala, Sweden.
4. Department of Surgery, Umeå University, Umeå, Sweden
5. Department of Internal Medicine, Krefting Research Center, Sahlgrenska Academy, University of Gothenburg, Gothenburg, Sweden
6. Occupational and Environmental Medicine, Department of Public Health and Clinical Medicine, Umeå University, Umeå, Sweden

Corresponding author

Christer Janson

Department of Medical Sciences: Respiratory, Allergy and Sleep Research, Uppsala University, 75185 Uppsala, Sweden.

christer.janson@medsci.uu.se

Tel +46704250441

Fax +46186110228

For peer review only

Abstract:

Objective: Habitual snoring is associated with fatigue, headaches, and low work performance. This cross sectional study aimed to investigate if snoring is affected by environmental factors such as home dampness and exposure to air pollution.

Setting: General population sample from four Swedish cities.

Participants: 25,848 participants from the Swedish part of the epidemiological GA2LEN study carried out in 2008. The participants completed a postal questionnaire on snoring and, indoor and outdoor environmental exposure as well as potential confounders including smoking, weight, height and educational level.

Results: Of the participants, 4,211 (16.3%) were habitual snorers. Habitual snorers reported water damage (8.3% vs. 7.0% $p<0.0001$), floor dampness (4.6% vs. 3.8%% $p<0.0001$) and visible mould (5.2% vs. 3.8% $p<0.0001$) in their homes more often than non-snorers. Habitual snorers stated being annoyed by air pollution more often than non-snorers with habitual snorers reporting being irritated with the air in their residential area to a higher extent (*sometimes* 16.2% vs.13.9%, and *daily* 4.6% vs.3.1%) as well as annoyance from traffic fumes (*somewhat* 19% vs.18.5% and *very* 5% vs 3.6%) ($p<0.0001$). These results remained significant after adjustment for age, BMI, smoking history and educational level.

Conclusion: Snoring is more prevalent in subjects reporting home dampness and air pollution. These association should be confirmed in further research using objective measurements and a longitudinal approach.

For peer review only - http://bmjopen.bmj.com/site/about/guidelines.xhtml

Strength and limitations

The sample size is large giving it considerate power and the possibility to simultaneously control for several confounding risk factors.

The association between snoring and both indoor and outdoor exposure has only been studied to a limited extent previously

The study is cross sectional and causality can therefore not been assessed.

The assessments of exposure are self-reported and we know that there is only a moderate association between self-reported and measured air pollution.

For peer review only

For peer review only - http://bmjopen.bmj.com/site/about/guidelines.xhtml

Introduction

Snoring is a sign of compromised upper airway and a common symptom in obstructive sleep apnoea.[1] Snoring is a health problem also in the absence of sleep apnoea, and studies in both adults and children show that it is the frequency of snoring rather than the apnoea hypopnea index that predicts symptoms and poorer behavioural and cognitive outcomes.[2, 3]

Habitual snoring is reported to occur in between 16-33% in men and 6-19% in women. [4-8] Male sex, age, obesity, smoking and nasal congestion are all well-established risk factors for habitual snoring.[9-14] An association between home dampness and insomnia symptoms was reported in a population-based cross-sectional study,[15] but only a few studies have, to our knowledge investigated the possible association between habitual snoring and building dampness [16].

Air pollutants such as particulate matter (PM), ozone (O_3) and nitrogen dioxide (NO_2) all have adverse effects on the airways. [17] An association between exposure to air pollutants and snoring have been reported in children [18, 19] and habitual snoring was associated with having a bedroom with traffic noise but not to high exposure to traffic pollution in a Nordic population study .[20] In another study, exposure to ozone was associated with an increase in AHI.[21]

The aim of this study was to investigate if habitual snoring is affected by environmental factors. We hypothesised that both exposures to perceived building dampness and air pollution are associated with habitual snoring.

For peer review only - http://bmjopen.bmj.com/site/about/guidelines.xhtml

Method

The Global Asthma and Allergy and European network of excellence (GA²LEN) was established 2004 as a consortium in the field of asthma and allergy.[22] The GA²LEN epidemiological study was carried out in 2008 across Europe, and the aim was to assess the prevalence of allergic disease and its associated risk factors. [23] The present investigation was based on the Swedish part of the postal survey carried out in four centres; Stockholm, Gothenburg, Uppsala and Umeå. A random sample of 15,000 subjects from Gothenburg and 10000 from each of the other cities was selected, and the participants were 16-75 years old.[24] The questionnaire included questions on asthma, chronic bronchitis, eczema, insomnia, OSA, physical activity and environmental and workplace exposure. Up to three reminders were mailed, and 26,577 subjects completed the questionnaire. In the present analysis, we included the 25,848 participants who had answered both the question regarding snoring and environmental exposure. Informed consent was obtained from each participant. The study was approved by the Regional Ethical Review Board in Uppsala, Sweden (Dnr 2008/014).

Definitions:

Snoring was assessed by the question, "in the last few months, how many times have you snored loudly and disturbingly". Habitual snorers were defined as having answered that they snored either 3-5 times per week or every or almost every night.

Smoking history was assessed by the questions "Have you ever smoked one or more cigarettes per day for more than one year?" Those that answered yes to that question were then asked: "Have you smoked at all during the last month?".

Never smoking was defined as a negative response to the question, "Have you ever smoked for at least one year?".

Previous smoking was defined as a positive response to the question "Have you ever smoked for at least one year?" and a negative response to the question "Have you smoked at all during the last month?".

Current smoking was defined as positive responses to the questions "Have you ever smoked for at least one year?" and "Have you smoked at all during the last month?".

Air pollution was assessed by two questions "Do you find the air irritating in your residential area: 1, Daily/almost daily (Daily) 2, Sometimes/periodically (Sometimes) 3, Seldom/never (Never) and "How annoyed are you by fumes from traffic in your residential area.": 1, None/a little (None) 2, Somewhat 3 Very much.

For peer review only - http://bmjopen.bmj.com/site/about/guidelines.xhtml

Dampness was defined as a positive response to at least one of the following questions: "Have any of the following been noted in your home during the last 12 months:

(a) Water-/moisture damage on indoor walls, floors or ceiling? (Water damage)

(b) Dented plastic mats, yellowed plastic mats or blackened parquet? (Floor dampness)

(c) Visible moulds on walls, floors or ceiling?" (Visible moulds)

Educational level was assessed, and the subjects were divided into three groups depending on the highest academic level: primary school, high school, university.

Body mass index (BMI) was calculated from the subjects answer to their height and weight.

Statistical Analysis:

The statistics where analysed using STATA version 15 (STATA Corp, College Station, Texas, USA). Chi-squared test and unpaired t-test were used in unadjusted analyses when comparing habitual snorers and non-snorers. Multiple logistic regression analysis was performed when studying the independent association between habitual snoring and environmental exposure. To decide which variables to adjust for in the multiple logistic regression models, a directed acyclic graph was used (http://www.dagitty.net) (Supplemental Figure 1). The confounding factors used in the model were age, BMI, smoking history and educational level. Test for interaction was done for building dampness and air pollution indicators in the relationship with snoring. We also used a test for interaction to determine if there was a sex difference in the association between snoring and environmental variables. A p-value of <0.05 was considered to be statistically significant.

Patient and public involvement

The European Federation of Allergy and Airways Diseases Patients' Associations is a partner of the Global Asthma and Allergy and European network of excellence. Apart from this no patients or members of the public were involved in the design, conduct, or dissemination plan for this study.

For peer review only - http://bmjopen.bmj.com/site/about/guidelines.xhtml

Results

Of the 25,848 participants 4,211 (16.3%) were habitual snorers. Habitual snoring was more common in men and in older participants (Table 1). Habitual snorers had a higher mean BMI and were less likely to have finished higher education, and were more likely to be ex- and current smokers than non-snorers (Table 1).

Habitual snorers reported water damage, floor dampness and visible mould more often than non-snorers. The habitual snorers also reported being exposed to air pollution more often than non-snorers (Table 1).

Residential water damage, floor dampness, and visible moulds were significantly associated with habitual snoring after adjusting for confounders (age, BMI, smoking history and educational level) (Table 2). There was also an association between reported exposures to residential air pollution and being exposed to traffic related air pollution and habitual snoring, respectively, that remained statistically significant after adjustment for confounders (Table 2).

There was no significant difference in the association between reported air pollution and habitual snoring in those that reported or did not report residential water damage. There was no significant difference between women and men in the associations between building dampness and air pollution with snoring (all $p_{interaction}$-values > 0.05).

For peer review only - http://bmjopen.bmj.com/site/about/guidelines.xhtml

For peer review only

Discussion

This study's main result was that both self-reported home dampness and exposure to air pollution were significantly associated with habitual snoring. The results remained significant after having adjusted for age, BMI, smoking history and educational level.

The results that habitual snoring was associated with both airway pollution and home dampness are important as previous studies have found that habitual snores have an increased risk of work-related accidents [25], daytime sleepiness [26], hypertension [27], diabetes [28] and increased mortality.[29]. Some of these associations are probably related to obstructive sleep apnea, but several studies have found that snoring, even in the absence of sleep apnea, is associated with daytime sleepiness.[3,30] These results further implicate the importance of good air quality in home and outdoor environment for health.

Of the indoor dampness indicators, visible mould had the highest odds ratio for habitual snoring, followed by water damage and floor dampness. As far as we know, a relationship between home dampness and snoring has only been in a few studies.[16] However, previous studies have shown associations between snoring and other respiratory conditions such as asthma and chronic rhinosinusitis. [31-33] Zhang et al. showed an association between building dampness and non-respiratory symptoms such as eye and skin symptoms, headache, and fatigue. [34] An association between indoor dampness and insomnia symptoms has also been reported. [15] Headache and fatigue are symptoms that are associated with snoring. [10, 35] It is therefore possible that the association between home dampness, fatigue and headache partly is related to a higher prevalence of habitual snoring in individuals living in damp homes.

In the present study, the level of annoyance was used as a proxy for the actual extent of pollution. The association between air pollution and snoring in our study is in accordance with previous research by Weinreich *et al.* [21], where there was an association between ozone levels and obstructive sleep apnea. In another study, children residing in the neighbourhoods with the poorest air quality had a significantly higher prevalence of habitual snoring than those who lived in the neighbourhoods with lower air pollution degrees.[18] Also, passive and active smoking has been associated with habitual snoring [13], and air pollution and cigarette smoke share some chemical components and mechanic pathways. [36]. However, our finding is not in line with the findings of Gislason et al. [20], were traffic noise but not high air pollution exposure was associated with snoring.

For peer review only - http://bmjopen.bmj.com/site/about/guidelines.xhtml

The prevalence of habitual snoring was 16% in our study sample. This is lower compared to a similar study on a Scandinavian population, which showed a prevalence of 25%. [20] As in many previous studies, habitual snoring was associated with older age, male sex, higher BMI and smoking. [9]

The biological explanation for the association between snoring and environmental exposure in our study is not completely known. Home dampness and exposure to airway pollution has been associated with inflammatory markers in nasal lavage.[37, 38] It is therefore possible that inflammation in the upper airways caused by home dampness and air pollution explains the association with snoring.

The study has a large sample size, thereby giving it considerate power and the possibility to simultaneously control for several confounding risk factors. However, there are weaknesses to be considered. The exposure assessments are self-reported, and we know that there is only a moderate association between self-reported and measured air pollution.[39] It is difficult to say how accurate the subjects' assessment of their snoring habits are, and it has been shown that there are some discrepancies between self-reported and spouse-reported snoring habits. [40] We also have no data on if the subjects live by themselves or not, a factor that may affect individuals knowledge of their snoring. The participation rate was 57%, and we know from previous analysis that younger persons and men are somewhat underrepresented in our population [41]. We do not, however, think that this has influenced the results in a major way.

In conclusion, home dampness and exposure to air pollution were significantly associated with habitual snoring also after adjusting for cofounders. These associations should be confirmed in further research using objective measurements and a longitudinal approach.

Acknowledgements

The study was carried out as part of the GA2LEN survey and follow-up and was supported by the EU FP6 project GA2LEN (EU contract nr. FOOD-CT-2004-506378).

For peer review only

References

1. Zhang, W. and L.Y. Si, *Obstructive sleep apnea syndrome (OSAS) and hypertension: pathogenic mechanisms and possible therapeutic approaches.* Ups J Med Sci, 2012. **117**(4): p. 370-82.
2. Smith, D.L., et al., *Frequency of snoring, rather than apnea-hypopnea index, predicts both cognitive and behavioral problems in young children.* Sleep Med, 2017. **34**: p. 170-178.
3. Svensson, M., et al., *Daytime sleepiness relates to snoring independent of the apnea-hypopnea index in women from the general population.* Chest, 2008. **134**(5): p. 919-924.
4. Enright, P.L., et al., *Prevalence and correlates of snoring and observed apneas in 5,201 older adults.* Sleep, 1996. **19**(7): p. 531-8.
5. Gislason, T., et al., *Prevalence of sleep apnea syndrome among Swedish men--an epidemiological study.* J Clin Epidemiol, 1988. **41**(6): p. 571-6.
6. Kayukawa, Y., et al., *Habitual snoring in an outpatient population in Japan.* Psychiatry Clin Neurosci, 2000. **54**(4): p. 385-91.
7. Lindberg, E., et al., *A 10-year follow-up of snoring in men.* Chest, 1998. **114**(4): p. 1048-55.
8. Svensson, M., *Risk Factors Associated With Snoring in Women With Special Emphasis on Body Mass Index: A Population-Based Study.* Chest, 2006. **129**(4): p. 933-941.
9. Bloom, J.W., W.T. Kaltenborn, and S.F. Quan, *Risk factors in a general population for snoring. Importance of cigarette smoking and obesity.* Chest, 1988. **93**(4): p. 678-83.
10. Janson, C., et al., *Daytime sleepiness, snoring and gastro-oesophageal reflux amongst young adults in three European countries.* J Intern Med, 1995. **237**(3): p. 277-85.
11. Palm, A., C. Janson, and E. Lindberg, *The impact of obesity and weight gain on development of sleep problems in a population-based sample.* Sleep Med, 2015. **16**(5): p. 593-7.
12. Wali, S.O. and B.A. Abaalkhail, *Prevalence and predictors of habitual snoring in a sample of Saudi middle-aged adults.* Saudi Med J, 2015. **36**(8): p. 920-7.
13. Franklin, K.A., et al., *The influence of active and passive smoking on habitual snoring.* Am J Respir Crit Care Med, 2004. **170**(7): p. 799-803.
14. Young, T., L. Finn, and M. Palta, *Chronic nasal congestion at night is a risk factor for snoring in a population-based cohort study.* Arch Intern Med, 2001. **161**(12): p. 1514-9.
15. Janson, C., et al., *Insomnia is more common among subjects living in damp buildings.* Occup Environ Med, 2005. **62**(2): p. 113-8.
16. Wang, J., et al., *Dampness and mold at home and at work and onset of insomnia symptoms, snoring and excessive daytime sleepiness.* Environ Int, 2020. **139**: p. 105691.
17. Guan, W.J., et al., *Impact of air pollution on the burden of chronic respiratory diseases in China: time for urgent action.* Lancet, 2016. **388**(10054): p. 1939-1951.
18. Kheirandish-Gozal, L., et al., *Neighbourhood air quality and snoring in school-aged children.* Eur Respir J, 2014. **43**(3): p. 824-32.
19. Sanchez, T., et al., *Association between air pollution and sleep disordered breathing in children.* Pediatr Pulmonol, 2019. **54**(5): p. 544-550.
20. Gislason, T., et al., *Self-reported exposure to traffic pollution in relation to daytime sleepiness and habitual snoring: a questionnaire study in seven North-European cities.* Sleep Med, 2016. **24**: p. 93-99.
21. Weinreich, G., et al., *Association of short-term ozone and temperature with sleep disordered breathing.* Eur Respir J, 2015. **46**(5): p. 1361-9.
22. Van Cauwenberge, P., et al., *Spreading excellence in allergy and asthma: the GA2LEN (Global Allergy and Asthma European Network) project.* Allergy, 2005. **60**(7): p. 858-864.
23. Jarvis, D., et al., *Asthma in adults and its association with chronic rhinosinusitis: the GA2LEN survey in Europe.* Allergy, 2012. **67**(1): p. 91-8.
24. Sundbom, F., et al., *Asthma symptoms and nasal congestion as independent risk factors for insomnia in a general population: results from the GA(2)LEN survey.* Allergy, 2013. **68**(2): p. 213-9.
25. Lindberg, E., et al., *Role of snoring and daytime sleepiness in occupational accidents.* Am J Respir Crit Care Med 2001, **164**(11): p. 2031-2035.
26. Theorell-Haglöw, J., et al., *What are the important risk factors for daytime sleepiness and fatigue in women?* Sleep 2006, **29**(6): p. 751-757.

For peer review only - http://bmjopen.bmj.com/site/about/guidelines.xhtml

27. Lindberg, E., et al., *Snoring and hypertension - a 10-year follow-up.* Eur Respir J 1998, **11**(4):p. 884-889.
28. Lindberg, E.,et al., *Snoring and daytime sleepiness as risk factors for hypertension and diabetes in women - a population-based study* Respir Med 2007, **101**(6):p. 1283-1290.
29. Lindberg, E., et al., *Snoring, daytime sleepiness and mortality - a prospective, population based study*. Thorax 1998, **53**(8):p. 631-637.
30. Janson, C., et al., *Excessive Daytime Sleepiness and fatigue in non-apnoeic snorers: improvement after UPPP.* Eur Respir J 1994, **7**(5): p. 845-849.
31. Ahlroth Pind, C., et al., *Patient-reported signs of dampness at home may be a risk factor for chronic rhinosinusitis: A cross-sectional study.* Clin Exp Allergy, 2017. **47**(11): p. 1383-1389.
32. Gunnbjornsdottir, M.I., et al., *Indoor environment in three North European cities in relationship to atopy and respiratory symptoms.* Clin Respir J, 2009. **3**(2): p. 85-94.
33. Wang, J., et al., *Dampness, mould, onset and remission of adult respiratory symptoms, asthma and rhinitis.* Eur Respir J, 2019. **53**(5).
34. Zhang, X., et al., *Dampness and mold in homes across China: Associations with rhinitis, ocular, throat and dermal symptoms, headache and fatigue among adults.* Indoor Air, 2019. **29**(1): p. 30-42.
35. Stark, C.D. and R.J. Stark, *Sleep and chronic daily headache.* Curr Pain Headache Rep, 2015. **19**(1): p. 468.
36. Ghio, A.J., J.M. Soukup, and M.C. Madden, *The toxicology of air pollution predicts its epidemiology.* Inhalation Toxicology, 2018. **30**(9-10): p. 327-334.
37. Chen, B.Y., et al., *The association of ambient air pollution with airway inflammation in schoolchildren.* Am J Epidemiol, 2012. **175**(8): p. 764-74.
38. Wieslander, G., et al., *Nasal and ocular symptoms, tear film stability and biomarkers in nasal lavage, in relation to building-dampness and building design in hospitals.* Int Arch Occup Environ Health, 1999. **72**(7): p. 451-61.
39. Carlsen, H.K., et al., *Indicators of residential traffic exposure: Modelled NOX, traffic proximity, and self-reported exposure in RHINE III.* Atmospheric Environment, 2017. **167**: p. 416-425.
40. Wiggins, C.L., et al., *Comparison of self- and spouse reports of snoring and other symptoms associated with sleep apnea syndrome.* Sleep, 1990. **13**(3): p. 245-52.
41. Jerning C, et al., *Asthma and physical activity e A population based study results from the Swedish GA2LEN survey.* Respir Med; 2013. **107**(11), p. 1651-1658

For peer review only

Contributors CJ is the corresponding author and DS and CJ drafted the paper and performed the statically analysis, CJ, RM, KF, BF and BL participated in designing the study and reviewed the manuscript; JT, ML, RM, JW, KF, DN. BL, BF and EL also reviewed the paper at several stages

Funding This work has been supported by supported by the EU FP6 project GA2LEN (EU contract nr. FOOD-CT-2004-506378), the Centre for Allergy Research at the Karolinska Institutet, the Swedish Heart Lung Foundation, the Swedish Heart and Lung Association and the Swedish Asthma and Allergy Association.

Competing interests None declared.

Patient and public involvement The European Federation of Allergy and Airways Diseases Patients' Associations is a partner of the Global Asthma and Allergy and European network of excellence. Apart from this no patients or members of the public were involved in the design, conduct, or dissemination plan for this study.

Patient consent for publication Not required.

Ethics approval The study was approved by the Regional Ethical Review Board in Uppsala, Sweden approval (Dnr 2008/014).

Data availability statement Data are available upon reasonable request. Requests for data should be sent to the corresponding author.

For peer review only

For peer review only - http://bmjopen.bmj.com/site/about/guidelines.xhtml

Table 1. Characteristics and environmental exposure in non-snorers and snorers (% and mean±SD)

	Non-snorers (n=21,637)	Habitual snores (n=4,211)	p-value
Men	42.3	62.6	<0.0001
Age	42.1±16.0	50.0±13.9	<0.0001
Smoking history			<0.0001
never	63.7	45.7	
ex	23.6	34.2	
current	12.7	20.1	
BMI	24.3±3.9	27.1±4.6	<0.0001
Educational level			<0.0001
Primary school,	12.4	18.5	
Secondary school	28.5	35.8	
Tertiary School	59.1	45.8	
Indoor dampness			
Water damage	7.0	8.3	0.002
Floor dampness	3.8	4.6	0.02
Visible moulds	3.8	5.2	<0.0001
Air pollution in residential area			<0.0001
Never	83.0	79.2	
Sometimes	13.9	16.2	
Daily	3.1	4.6	
Air pollution from traffic			<0.0001
None	77.9	76.0	
Somewhat	18.5	19.0	
Very much	3.6	5.0	

Table 2. Association between environmental exposure and habitual snoring.

	Odds ratio (95% confidence interval)	p-value
*Indoor dampness**		
Water damage	1.29 (1.13-1.48)	<0.0001
Floor dampness	1.21 (1.01-1.45)	0.04
Visible moulds	1.51 (1.27-1.80)	<0.0001
*Air pollution in residential area***		
Never	1	
Sometimes	1.29 (1.13-1.48)	<0.0001
Daily	1.64 (1.36-1.98)	<0.0001
*Air pollution from traffic**c*		<0.0001
None	1	
Somewhat	1.15 (1.04-1.26)	0.004
Very much	1.51 (1.27-1.80)	<0.0001

* adjusted for age, BMI, smoking history, educational level and residential air pollution
** adjusted for age, BMI, smoking history, educational level and residential water damage

For peer review only - http://bmjopen.bmj.com/site/about/guidelines.xhtml

For peer review only

Supplemental figure 1. Directed acyclic graph of the association between snoring and environmental exposure. Green arrows indicate causal paths, while red arrows show biasing paths. http://www.dagitty.net

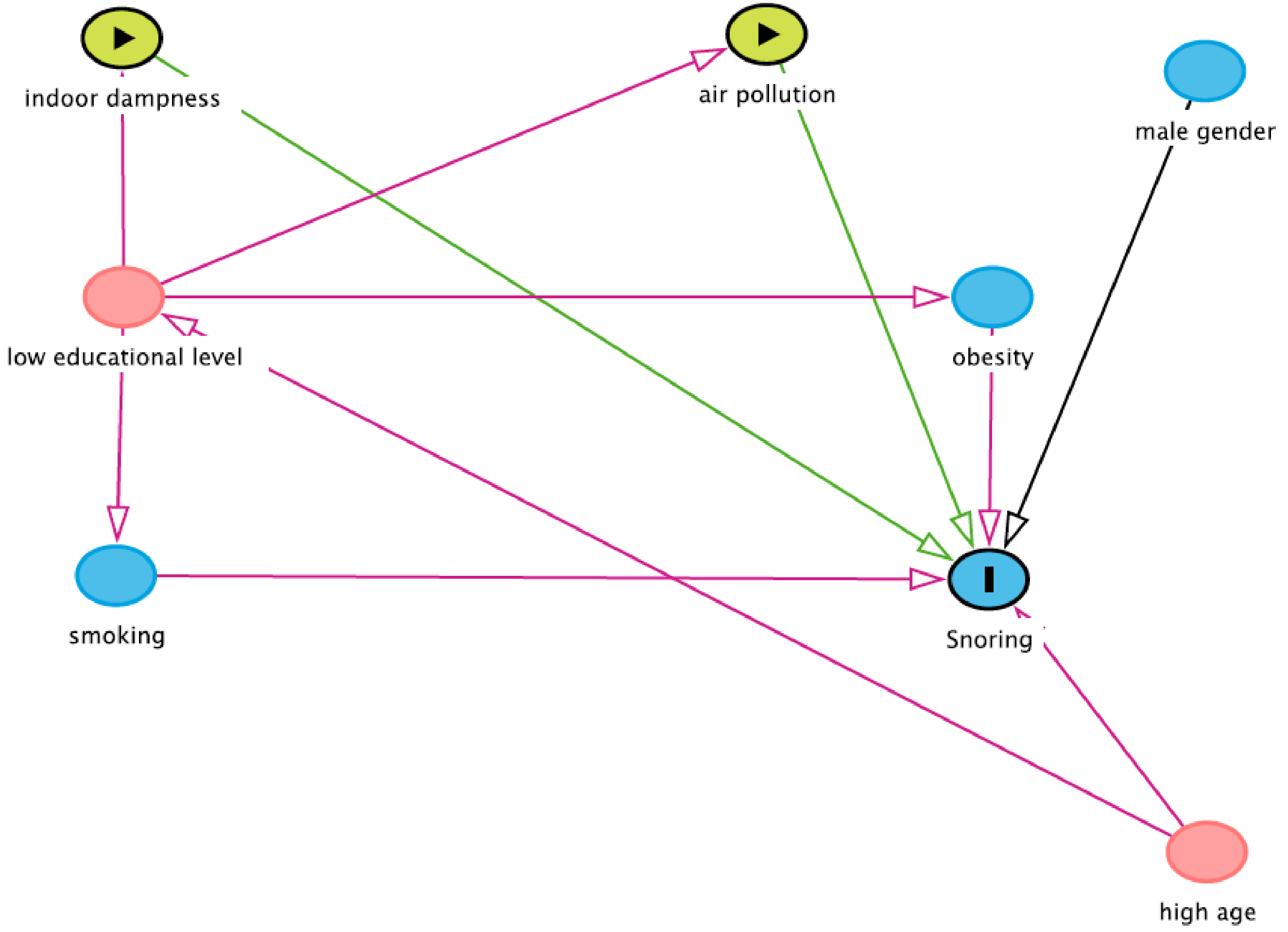

STROBE Statement—Checklist of items that should be included in reports of ***cross-sectional studies***

	Item Snoring and environmental exposure: results from the Swedish GA2LEN study No	Recommendation
**Title and abstract**	1	(*a*) Indicate the study's design with a commonly used term in the title or the abstract<br>Page 2, paragraph 1
		(*b*) Provide in the abstract an informative and balanced summary of what was done and what was found
**Introduction**		
Background/rationale	2	Explain the scientific background and rationale for the investigation being reported<br>Page 4, paragraph 1-3
Objectives	3	State specific objectives, including any prespecified hypotheses<br>Page 4, paragraph 4
**Methods**		
Study design	4	Present key elements of study design early in the paper<br>Page 5, paragraph 1
Setting	5	Describe the setting, locations, and relevant dates, including periods of recruitment, exposure, follow-up, and data collection<br>Page 5, paragraph 1
Participants	6	(*a*) Give the eligibility criteria, and the sources and methods of selection of participant<br>Page 5, paragraph 1s
Variables	7	Clearly define all outcomes, exposures, predictors, potential confounders, and effect modifiers. Give diagnostic criteria, if applicable<br>Page 5, paragraph 2- page 6, paragraph 3
Data sources/ measurement	8*	For each variable of interest, give sources of data and details of methods of assessment (measurement). Describe comparability of assessment methods if there is more than one group<br>Page 5, paragraph 2- page 6, paragraph 3
Bias	9	Describe any efforts to address potential sources of bias<br>Not relevant
Study size	10	Explain how the study size was arrived at<br>Page 5, paragraph 1
Quantitative variables	11	Explain how quantitative variables were handled in the analyses. If applicable, describe which groupings were chosen and why<br>Page 6, paragraph 4
Statistical methods	12	(*a*) Describe all statistical methods, including those used to control for confounding<br>Page 6, paragraph 4
		(*b*) Describe any methods used to examine subgroups and

For peer review only - http://bmjopen.bmj.com/site/about/guidelines.xhtml

		interactions<br>Page 6, paragraph 4
		(*c*) Explain how missing data were addressed
		(*d*) If applicable, describe analytical methods taking account of sampling strategy
		(*e*) Describe any sensitivity analyses
**Results**		
Participants	13*	(a) Report numbers of individuals at each stage of study—eg numbers potentially eligible, examined for eligibility, confirmed eligible, included in the study, completing follow-up, and analysed<br>Page 5, paragraph 1 and Page 7, paragraph 1
		(b) Give reasons for non-participation at each stage<br>Page 5, paragraph 1
		(c) Consider use of a flow diagram<br>Will add little of value in this paper
Descriptive data	14*	(a) Give characteristics of study participants (eg demographic, clinical, social) and information on exposures and potential confounders<br>Table 1
		(b) Indicate number of participants with missing data for each variable of interest
Outcome data	15*	Report numbers of outcome events or summary measures<br>Page 5, paragraph 1
Main results	16	(*a*) Give unadjusted estimates and, if applicable, confounder-adjusted estimates and their precision (eg, 95% confidence interval). Make clear which confounders were adjusted for and why they were included<br>Table 2, Figure 1, Page 7, paragraph 3
		(*b*) Report category boundaries when continuous variables were categorized<br>Not relevant
		(*c*) If relevant, consider translating estimates of relative risk into absolute risk for a meaningful time period<br>Not relevant
Other analyses	17	Report other analyses done—eg analyses of subgroups and interactions, and sensitivity analyses<br>Page 7, paragraph 4
**Discussion**		
Key results	18	Summarise key results with reference to study objectives<br>Page 8, paragraph 1
Limitations	19	Discuss limitations of the study, taking into account sources of potential bias or imprecision. Discuss both direction and magnitude of any potential bias<br>Page 9, paragraph 3
Interpretation	20	Give a cautious overall interpretation of results considering objectives, limitations, multiplicity of analyses, results

For peer review only

For peer review only - http://bmjopen.bmj.com/site/about/guidelines.xhtml

		from similar studies, and other relevant evidence<br>Page 9, paragraph 4 and 5
Generalisability	21	Discuss the generalisability (external validity) of the study results<br>Page 9, paragraph 4 and 5
**Other information**		
Funding	22	Give the source of funding and the role of the funders for the present study and, if applicable, for the original study on which the present article is based<br>Page 13

*Give information separately for exposed and unexposed groups.

Note: An Explanation and Elaboration article discusses each checklist item and gives methodological background and published examples of transparent reporting. The STROBE checklist is best used in conjunction with this article (freely available on the Web sites of PLoS Medicine at http://www.plosmedicine.org/, Annals of Internal Medicine at http://www.annals.org/, and Epidemiology at http://www.epidem.com/). Information on the STROBE Initiative is available at www.strobe-statement.org.

For peer review only
